# Effects of Preventive Nutrition Interventions among Adolescents on Health and Nutritional Status in Low- and Middle-Income Countries: A Systematic Review and Meta-Analysis

**DOI:** 10.3390/nu12010049

**Published:** 2019-12-23

**Authors:** Rehana A Salam, Jai K Das, Wardah Ahmed, Omar Irfan, Sana Sadiq Sheikh, Zulfiqar A Bhutta

**Affiliations:** 1 Division of Women and Child Health, Aga Khan University Hospital, Karachi 74800, Pakistan; rehana.salam@aku.edu (R.A.S.); jai.das@aku.edu (J.K.D.); wardah_ahmed93@hotmail.com (W.A.);; 2 Centre for Global Child Health, the Hospital for Sick Children, Toronto, ON M5G 0A4, Canada; omarirfan1@hotmail.com

**Keywords:** adolescent health, nutrition, nutrition interventions

## Abstract

The objective of this review was to assess the impact of preventive nutrition interventions on health and nutritional status of adolescents aged 10–19 years in low- and middle-income countries (LMICs). We searched the databases until 5 February 2019 without any restrictions on publication, date, language, or publication status. A total of 10 studies (15 papers) including 10,802 participants assessing the impact of micronutrient supplementation/fortification were included in this review. We did not find any study assessing the impact of nutrition education and counseling or macronutrient supplementation among adolescents. Among primary outcomes, we are uncertain of the effect of iron supplementation with or without folic acid on anemia (daily supplementation; relative risk (RR): 1.04, 95% confidence interval (CI) 0.42, 2.57; one study; 1160 participants; low-quality evidence; weekly supplementation; RR: 1.07, 95% CI: 0.46, 2.52; one study; 1247 participants; low-quality evidence). We are also uncertain of the effect of various micronutrient supplementation/fortification on body mass index (BMI) (calcium/vitamin D supplementation; (MD: −0.01 kg/m^2^; 95% CI: −1.20, 1.17; two studies; 730 participants; I^2^ 94%; very-low-quality evidence, iron supplementation with or without folic acid; MD: 0.47 kg/m^2^; 95% CI: −0.17, 1.11; two studies; 652 participants; I^2^ 37%; very-low-quality evidence, zinc supplementation; MD: 0.35 kg/m^2^; 95% CI: −0.15, 0.85; one study; 382 participants; very-low-quality evidence) and multiple micronutrient (MMN) fortification; MD: 0.23 kg/m^2^, 95% CI: −0.11, 0.57; two studies; 943 participants; I^2^ 22%; very-low-quality evidence). None of the included studies reported any other primary outcomes including morbidity or adverse effects. Among secondary outcomes, iron supplementation with or without folic acid may improve hemoglobin concentrations, and calcium/vitamin D supplementation may improve serum 25(OH)D levels, while calcium only supplementation and calcium and vitamin D supplementation may marginally improve total body bone mineral density (BMD). We are uncertain of the effect of MMN fortification on hemoglobin concentrations, calcium supplementation on total body bone mineral content (BMC), calcium + vitamin D supplementation on total body BMC, and zinc supplementation on zinc levels. There is limited evidence of micronutrient supplementation/fortification among adolescents, especially adolescent boys, on health and nutritional status in LMICs. These findings should be interpreted with caution due to the low quality and limited number of studies.

## 1. Introduction

Adolescents (aged 10 to 19 years) account for about 16% of the global population, and about 90% of the adolescents live in low- middle- income countries (LMICs) [1,2,3]. It is a critical age group since many of the risk factors for adult non-communicable diseases develop during adolescence, and targeting adolescents with health interventions can have a positive ripple effect. Despite the decreasing trend in communicable, maternal, neonatal, and nutritional diseases, malnutrition (including undernutrition, micronutrient deficiencies, and overweight/obesity) still remains a major public health concern [4]. Moreover, the progress is inequitable since countries with a low and low–middle social development index (SDI) bear a higher burden of morbid children and adolescents compared to middle-, high–middle-, and high-SDI countries [4]. Iron deficiency anemia associated with other micronutrient deficiencies still accounts for over 2500 disability-adjusted life-years (DALYs) per 100,000 adolescents [1,2,3]. Iodine and vitamin A deficiencies are also reported to be prevalent among adolescents, especially in countries with low SDI [5].

Nutrition plays a critical role in transitioning from adolescence to healthy adults. Malnutrition among children and adolescents is associated with delayed growth, impaired cognitive maturation, lower intellectual quotient (IQ), behavioral problems, and increased risk of contracting communicable diseases [6]. Stunting (defined as height for age <−2 standard deviations of the World Health Organization (WHO) Child Growth Standards median) is a determinant of sub-optimal growth, childhood and adult health, learning capacity, and productivity [7]. Undernutrition has various determining factors including poverty, food insecurity, poor sexual and reproductive health, violence, and many infectious and non-infectious diseases [8]. Quality of the available foods and food insecurity are added challenges faced by adolescents in LMICs and limit the access to a variety of foods including meat, fruits, and vegetables [2,9]. The food choices and preferences of adolescents also impact malnutrition in some settings, despite adequate food access [10,11]. Adolescents globally are consuming less than adequate amounts of fruits and vegetables and alarmingly high levels of sodium and sugar [10,11]. Findings from a systematic review assessing dietary intake and practices among adolescent girls in LMICs suggested that less than half of girls reported eating dairy, meats, fruits, and vegetables. Moreover, the ones who consumed fruits and vegetables daily did not meet the World Health Organization (WHO) dietary guidelines [11].

Interventions like nutrition education and counseling [12,13,14,15], micronutrient supplementation [16], food fortification [1,17], and macronutrient supplementation [18] are advocated for improving nutritional status. There are existing systematic reviews assessing the impact of nutrition intervention among adolescents; however, these reviews were either not comprehensive (assess a single intervention or a specific micronutrient), had overlapping age groups (included children and youths along with adolescents), or were focused on female adolescents only [19,20]. Moreover, to our knowledge, there are no existing systematic reviews assessing the effect of nutrition education and counseling in this age group. This review aims to comprehensively evaluate the effectiveness of all the above mentioned preventive nutrition interventions in combination or alone. There is increasing evidence that health initiatives require health systems that can deliver services equitably and efficiently; thus, many global health initiatives now involve health system strengthening measures into their programs [21]. However, the majority of existing systematic reviews restricted their inclusion criterion to randomized trials without focusing on various contextual factors that might potentially impact the effect of nutrition interventions in this age group. We aimed to include large-scale program evaluations that were implemented in multiple communities targeting adolescents with the abovementioned preventive nutrition interventions. We also aimed to assess various contextual factors that might potentially influence the effectiveness of these nutrition interventions in this age group. This contextual information is based on the WHO health system building blocks framework describing health systems in terms of six core components: service delivery, health workforce, health information systems, access to essential medicines/supplies, financing, and leadership/governance [21].

The protocol for this review was published with the Campbell Collaboration at https://onlinelibrary.wiley.com/doi/10.1002/CL2.195.

## 2. Materials and Methods

### 2.1. Objective

The objective of this review was to assess the impact of preventive nutrition interventions (including nutrition education and counseling, micronutrient supplementation/fortification, and macronutrient supplementation) on improving the health and nutritional status of adolescents aged 10–19 years in LMICs. We also aimed to collate evidence regarding the various contextual factors based on the WHO health system building blocks framework that might potentially impact the effectiveness of these interventions in this age group.

### 2.2. Type of Studies and Participants

We included primary studies and large-scale program evaluations, using experimental and quasi-experimental designs that allowed for causal inference. We included randomized controlled trials (RCTs) (both cluster- and individual-level randomization), quasi-experimental studies with non-random assignment to intervention and comparison groups, controlled before–after studies (CBA), and interrupted time series (ITS). The target population was adolescents between 10 and 19 years of age from LMICs. We classified LMICs according to the World Bank criteria. We excluded studies conducted among hospitalized adolescents and adolescents with any pre-existing diseases and health conditions. Studies including only a subset of eligible participants were included only if the results provided information for the relevant subgroup separately (i.e., 10–19 years subgroup). The following interventions alone or in any combination were reviewed:

- Nutrition education and counseling: (refers to education aimed at promoting a healthy diet by increasing the diversity and amount of foods consumed through various platforms including schools, communities, peer-based networks, and computer- and web-based education).
- Micronutrient supplementation (refers to the provision of individual or mixture of nutrients separately from the diet in form of injections, tablets, capsules, syrups/liquids, or powders) and fortification (refers to the process in which micronutrients are added to processed foods).
- Macronutrient supplementation (refers to supplementary feeding, balanced energy and protein supplementation, and lipid-based nutrition supplementation (LNS)).

### 2.3. Type of Outcomes

We included all the studies that met our inclusion criteria, but only those studies that had the outcomes defined below were included in the quantitative synthesis. The primary outcomes included anemia (hemoglobin concentrations less than 11 g/dL), body mass index (BMI) (defined as weight in kilograms (kg) divided by height in meters squared), morbidity (any morbidity as reported by the study authors, e.g., infectious diseases, night blindness etc.), and adverse events (as reported by study authors). The secondary outcomes included hemoglobin concentration, micronutrient status, body composition, development outcomes (as reported by authors; could include cognitive development, interpersonal development, and social development), and all-cause mortality.

### 2.4. Search Methods

The search cut-off date was 5 February 2019 in the following electronic databases: Cochrane Controlled Trials Register (CENTRAL), MEDLINE, EMBASE, CINAHL, PsycINFO, the WHO nutrition databases, CAB Global Health, Social Science Citation Index, Scopus, WHO Global Health Index, ADOLEC, EPPI, and clinical trials registry (search strategies can be found in Appendix A). We searched Google Scholar along with key nutrition agencies database such as Nutrition International, the Global Alliance for Improved Nutrition, the World Food Program, and Harvest-Plus to search for non-indexed, gray literature to locate relevant program evaluations and any additional trials. We did not apply any restrictions based on publication date, language, or publication status. References of included articles, relevant reviews, and annotated bibliographies were scanned for eligible studies. We ran citation searches of included studies in Google Scholar to identify any recent studies missed from the database searches.

### 2.5. Data Collection and Analysis

Two reviewers screened titles and abstracts in duplicate. We retrieved the full text of all studies which passed this first level of screening. The full-text review was also done in duplicate. Disagreements were resolved by consultation with a third reviewer, and agreement on discordant decisions was reached by consensus. We collated multiple reports of the same study, so that each study rather than each report was the unit of interest in the review. We extracted data on study background, population and setting, intervention group details, comparison group details, outcomes, and other information. In addition, we also collected details based on the WHO health system building blocks framework describing health systems in terms of six core components including service delivery, health workforce, health information system, access to essential medicines and supplies, financing, and leadership [21]. We performed the meta-analysis using RevMan 5 [22]. For dichotomous data, we used odds ratios (OR) and risk ratios (RR) with 95% confidence intervals (CI). For continuous data, we used the mean difference (MD) with 95% CI, if outcomes were measured in the same way between trials. We used the standardized mean difference (SMD) with 95% CI to combine trials that measured the same outcome but used different methods of measurement. We assessed heterogeneity among studies in two ways. Firstly, we assessed heterogeneity at face value: heterogeneity in population, interventions, or outcomes. We used I^2^, Q, and tau^2^ statistics as a guide to assess heterogeneity, along with a visual inspection of forest plots. If multi-arm studies were included, we combined intervention groups or separated them into different forest plots, ensuring that there was no double-counting of participants.

Based on the availability of the data, we planned to conduct sub-group analysis for the following subgroups:

- Duration or intensity of intervention (e.g., short versus long term, one-off versus multiple sessions).
- Individual context versus group context (for nutrition education and counseling only, i.e., children receiving the intervention individually versus those in groups)
- Study setting: school, community, clinic, etc.
- Sex: male and female
- Population (e.g., urban population versus rural population; resource-poor versus resource-rich population)
- We also attempted to conduct subgroup analysis based on the WHO health system building blocks factors (where data was available).

However, since very few studies were included in each comparison within the review, we could not conduct any of the afore-mentioned subgroup analysis. We did, however, sub-group the outcomes according to the specific micronutrients being supplemented under the comparison of “micronutrient supplementation/fortification” for clarity.

### 2.6. Quality Assessment

For RCTs, we used the Cochrane risk of bias tool [23] which assesses selection bias, performance bias, detection bias, attrition bias, and reporting bias. We rated each component as “high”, “low”, or “unclear” for each risk of bias component. For non-randomized studies, we used the Cochrane Effective Practice and Organization of Care (EPOC) risk of bias criteria (based on additional criteria including similar baseline outcome measurements, similar baseline characteristics, knowledge of the allocated interventions adequately prevented during the study, protection against contamination, intervention independent of other changes, shape of intervention effect pre-specified, and intervention unlikely to affect data collection) and rated the studies as low risk, high risk or unclear risk [24]. We provided supporting evidence for the risk of bias judgements. Two independent reviewers performed quality appraisal for each study, and disagreements were resolved by discussion or consultation with a third reviewer.

We incorporated quality appraisal into result interpretation. We summarized the quality of evidence according to the outcomes as per the Grading of Recommendations, Assessment, Development, and Evaluation (GRADE) criteria [25]. A grade of “high”, “moderate”, “low” and “very low” was used for grading the overall evidence indicating the strength of an effect on specific health outcome based on methodological flaws within the component studies, consistency of results across different studies, generalizability of research results to the wider patient base, and how effective the treatments were shown to be [26]. For non-randomized studies, the evidence quality was upgraded based on large magnitude of effect, dose–response relationship, and effect of all plausible confounding factors in reducing the effect (where an effect is observed) or suggesting a spurious effect (when no effect is observed). Two reviewers discussed ratings and reached consensus, and disagreements were resolved by consulting a third reviewer. We developed a summary of findings to show the effects for the primary outcomes.

## 3. Results

### 3.1. Results of the Search

**Figure 1:**
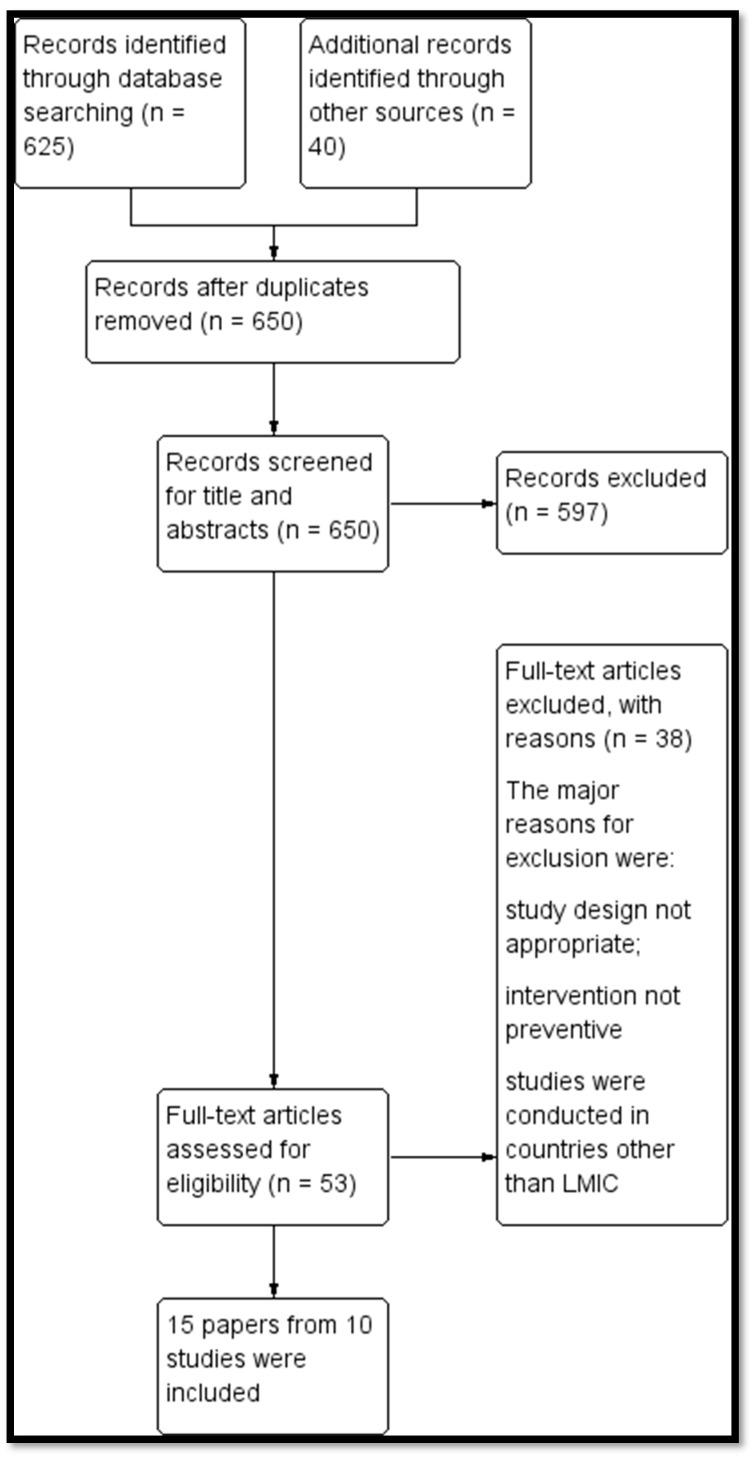
Search flow diagram.

We identified a total of 665 potentially relevant titles from the search. After removing duplicates, we screened 650 records for eligibility and excluded 597 articles on the basis of titles and abstracts. We obtained the full-text reports of the remaining 53 records and, of these, excluded 38 and included 15 papers (10 studies) in the review. Figure 1 depicts the search flow diagram, and the reasons for exclusion are provided in Appendix B. Table S1 provides the PRISMA checklist.

### 3.2. Description of Included Studies

This review includes 15 papers from 10 studies including 10, 802 participants [27,28,29,30,31,32,33,34,35,36,37,38,39,40,41]. All the studies were RCTs. All of the studies were conducted between 2003 and 2012 in LMICs including China [34], India [27,28,30,32,33], Sri Lanka [31], Bangladesh [35], and Indonesia [29,36]. These studies were all conducted in school settings. The majority of the studies (eight out of 10 studies) included adolescent girls aged between 10 and 19 years of age, while two studies [31,36] included both boys and girls. We did not find any study assessing nutrition education and counseling or macronutrient supplementation. All of the included studies provided micronutrient supplementation/fortification (any micronutrient alone or in combination). Among the micronutrient supplementation/fortification studies, two studies [32,34] provided calcium/vitamin D supplementation/fortification, four studies [27,29,31,33] provided iron supplementation with or without folic acid, two studies [28,31] provided zinc supplementation, one study [36] provided vitamin A supplementation, and three studies assessed MMN fortification [28,30,35]. The duration of intervention ranged from a minimum of 10 weeks [28] to a maximum of two years of intervention [34].

Among primary outcomes, the included studies reported anemia and BMI, while, among secondary outcomes, hemoglobin concentrations, micronutrient status (zinc, vitamin A, and vitamin D levels), body composition (total body BMC and total body BMD), and developmental outcomes were reported. None of the included studies reported morbidity and adverse effects among the primary outcomes and all-cause mortality among the secondary outcomes. We could not pool the outcomes for one study since it reported outcomes for pre-pubertal and post-pubertal girls and boys separately for all the intervention arms. The characteristics of the included studies are specified in Table 1.

### 3.3. Contextual Factors Based on WHO Health System Building Blocks

All the included studies were RCTs, and we did not find any large-scale nutrition intervention programs targeting adolescents from LMICs. We narratively synthesized the findings from the six health system building blocks based on the WHO health system building blocks framework (Table 2).

The service delivery platform in all of the included studies was a school, and the nutrition intervention in each study was delivered at a school. The nutrition interventions in five studies [29,31,32,33,35] were delivered through school teachers and student class monitors working with the study investigators. In one study [36], the intervention was delivered through field workers. Four studies [27,28,30,34] did not clearly specify the workforce utilized for the nutrition intervention delivery; however, from the description, it appeared that the intervention was probably delivered through school teachers. None of the included studies specified the details pertaining to health information systems. In all of the included studies, the nutrition supplement was provided by the researcher. Financing was provided by various not-for-profit organizations including UNICEF, Micronutrient Initiative, Zensar Foundation, SEAMEO-TROPMED Regional Center for Community Nutrition, University Grants Commission, International Atomic Energy Agency, Australian Dairy Research and Development Corporation, and Murray Goulburn Co-operative Co. One study [32] did not specify the financing, while there was no funding for one study [33]. In all of the included studies, study investigators led the intervention.

### 3.4. Risk of Bias

**Figure 2:**
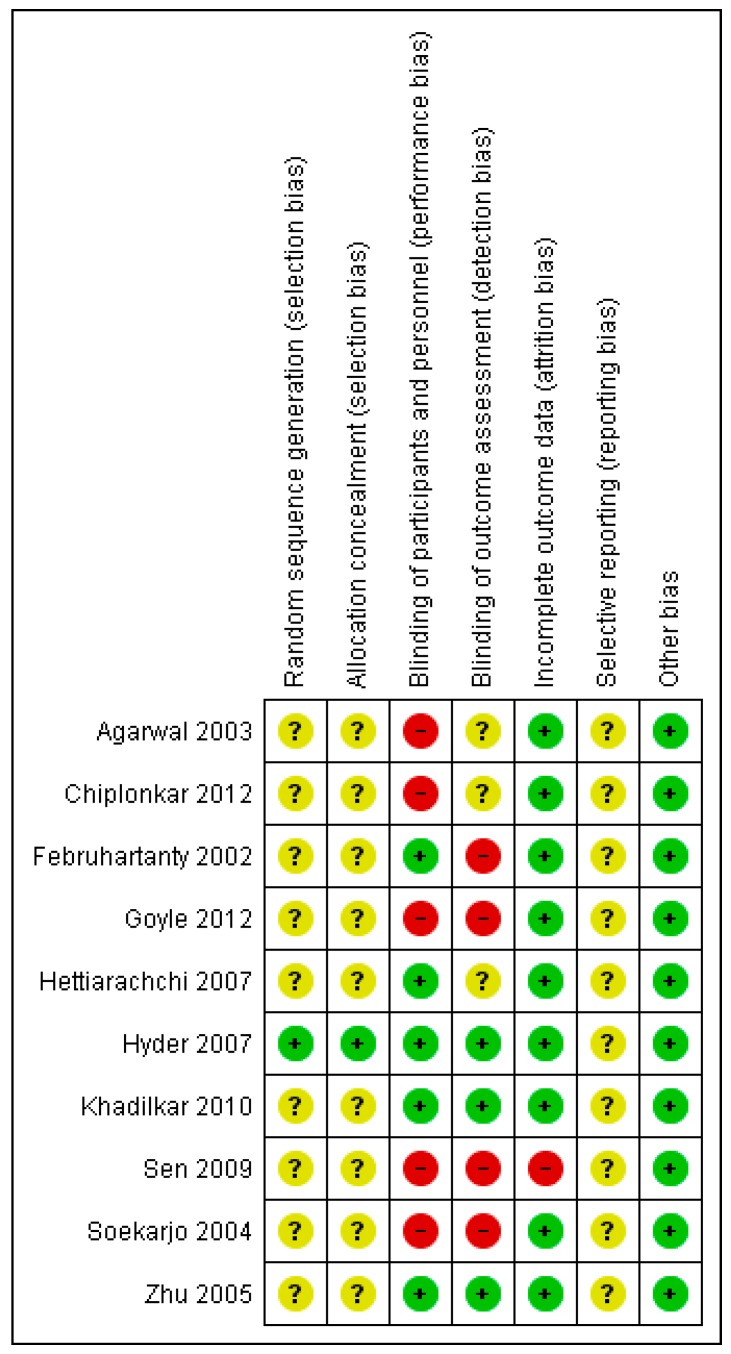
Risk of bias summary.

Overall, the included studies were judged to be at unclear risk of bias due to insufficient information regarding sequence generation, allocation concealment, and selective reporting. The majority of the studies lacked blinding and were judged to be at high risk or unclear risk for blinding. The majority of the studies were at low risk of bias for incomplete outcome data and other biases. The summary of the risk of bias across the included studies is shown in Figure 2.

### 3.5. Effects of Intervention

#### 3.5.1. Comparison 1: Nutrition Education and Counseling

We did not find any study assessing the impact of nutritional education and counseling alone on health and nutritional status among adolescents in LMICs.

#### 3.5.2. Comparison 2: Micronutrient Supplementation and Fortification (Any Micronutrient Alone or in Combination)

A total of 15 papers from 10 studies including 10,802 participants assessed the impact of micronutrient supplementation/fortification. Two studies [32,34] assessed calcium/vitamin D supplementation/fortification, four studies [27,29,31,33] assessed iron supplementation with or without folic acid, two studies [28,31] assessed zinc supplementation, and one study [36] assessed vitamin A supplementation, while three studies assessed MMN fortification [28,30,35]. Two of the studies had multiple intervention arms and were included in multiple comparison groups. One study [28] provided an MMN fortified snack in one group and zinc supplement in the other group, while one study [31] provided an iron supplement in one group and a zinc supplement in the other group.

**Figure 3:**
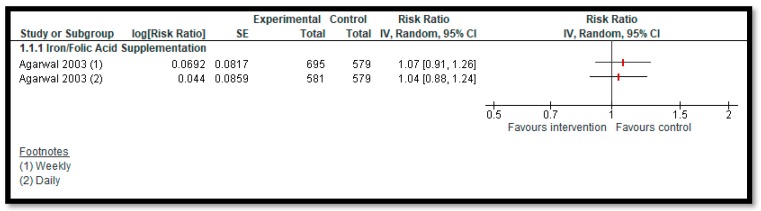
Impact of iron supplementation with or without folic acid on anemia.

**Figure 4:**
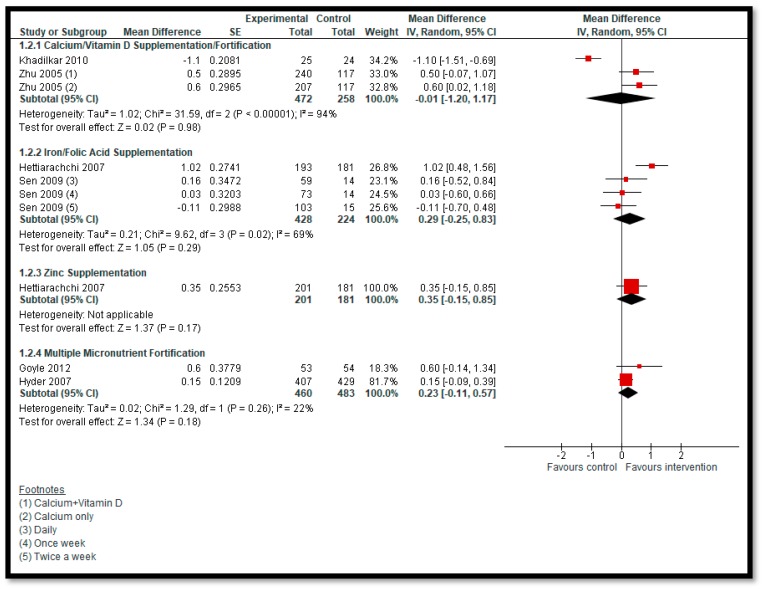
Impact of micronutrient supplementation/fortification on body mass index (BMI).

**Primary outcomes**: One study [27] reported on anemia. We are uncertain of the effect of daily (RR: 1.04, 95% CI 0.88, 1.24; one study; 1160 participants; low-quality evidence) or weekly supplementation (RR: 1.07, 95% CI: 0.91, 1.26; one study; 1274 participants; low-quality evidence) of iron supplementation with or without folic acid among adolescents on anemia (Figure 3). We are uncertain of the effect of calcium/vitamin D supplementation (MD: −0.01; 95% CI: −1.20, 1.17; two studies; 730 participants; I^2^ 94%; very-low-quality evidence), iron supplementation with or without folic acid (MD: 0.29; 95% CI: −0.25, 0.83; two studies; 652 participants; I^2^ 69%; very-low-quality evidence), zinc supplementation (MD: 0.35; 95% CI: −0.15, 0.85; one study; 382 participants; very-low-quality evidence), or MMN fortification (MD: 0.23, 95% CI: −0.11, 0.57; two studies; 943 participants; I^2^ 22%; very-low-quality evidence) on BMI among adolescents compared to no supplementation/fortification (Figure 4). None of the included studies reported on any other primary outcome, including morbidity or adverse effects.

**Figure 5:**
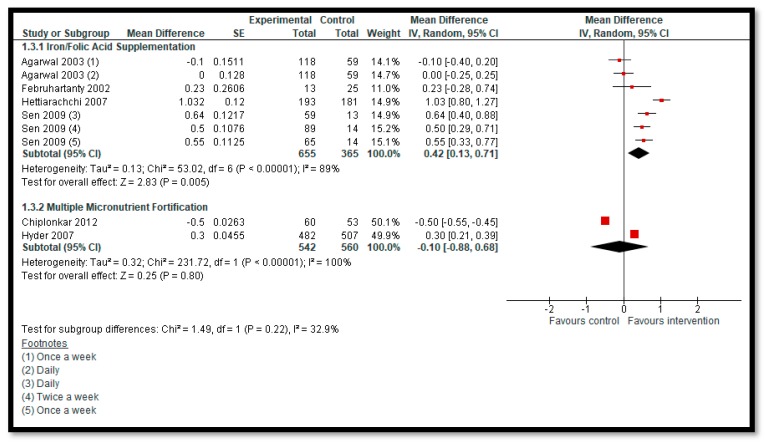
Impact of micronutrient supplementation/fortification on hemoglobin.

**Secondary outcomes**: Iron supplementation with or without folic acid may improve hemoglobin among adolescents when compared to no supplementation (MD: 0.42, 95% CI 0.13, 0.71; four studies; 1020 participants; I^2^ 89%; low-quality evidence) (Figure 5). We are uncertain of the effect of MMN fortification on hemoglobin when compared to no fortification (MD: −0.10, 95% CI: −0.88, 0.68; two studies; 1102 participants; I^2^ 100%; low-quality evidence). Calcium/vitamin D supplementation may improve serum 25(OH) D levels (SMD: 2.85, 95% CI: 0.89, 4.82; two studies; 395 participants; I^2^ 99%; low-quality evidence). We are uncertain of the effect of zinc supplementation on serum zinc levels (MD: 6.94, 95% CI: −4.84, 18.71; two studies; 494 participants; I^2^ 99%; low-quality evidence). We are uncertain of the effect of calcium only supplementation (MD: 30.20, 95% CI: −40.56, 100.96; one study; 233 participants; low-quality evidence) and calcium + vitamin D supplementation (MD: 21.60, 95% CI: −45.32, 88.52; one study; 235 participants; low-quality evidence) on total body BMC. We are uncertain of the effect of calcium only supplementation (MD: 0.02, 95% CI: −0.00, 0.04; one study; 233 participants; low-quality evidence) and calcium + vitamin D supplementation (MD: 0.02, 95% CI: −0.00, 0.04; one study; 235 participants; low-quality evidence) on total body BMD. One study, by Sen (2009), reported the impact of iron supplementation with or without folic acid on the cognition of adolescent girls, suggesting improved cognition in most of the tests with daily of twice weekly supplementation compared to once weekly or no supplementation. None of the other secondary outcomes including other development outcomes and all-cause mortality were reported.

#### 3.5.3. Comparison 3: Macronutrient Supplementation

We did not find any study assessing the impact of macronutrient supplementation on health and nutritional status among adolescents.

## 4. Discussion

This review summarizes findings from a total of 10 studies from 15 papers including 10, 802 participants. The findings are summarized in the summary of findings (Table 3). All the studies included in this review were RCTs and assessed the impact of micronutrient supplementation/fortification on health and nutritional status among adolescents in LMIC. We did not find any study assessing the impact of nutrition education and counseling or macronutrient supplementation. Micronutrient supplementation/fortification interventions included calcium/vitamin D supplementation/fortification, iron supplementation with or without folic acid, zinc supplementation, and MMN fortification. Among primary outcomes, we are uncertain of the effect of either daily or weekly supplementation of iron supplementation with or without folic acid on anemia. We are also uncertain of the effect of calcium/vitamin D supplementation, iron supplementation with or without folic acid, zinc supplementation, and MMN fortification on BMI among adolescents compared to no supplementation/fortification. None of the included studies reported any other primary outcome including morbidity or adverse effects. Among secondary outcomes, iron supplementation with or without folic acid may improve hemoglobin, and calcium/vitamin D supplementation may improve serum 25(OH) D levels, while calcium only supplementation and calcium + vitamin D supplementation may marginally improve total body BMD. We are uncertain of the effect of MMN fortification on hemoglobin, calcium supplementation on total body BMC, calcium + vitamin D supplementation on total body BMC, and zinc supplementation on zinc levels. One study reported the impact of iron supplementation with or without folic acid on the cognition of adolescent girls, suggesting improved cognition in most of the tests with daily or twice weekly supplementation compared to once weekly or no supplementation. The quality of the outcomes was either low or very low. The outcome quality was downgraded due to study limitations, including unclear sequence generation and allocation concealment methods and lack of blinding, as well as high heterogeneity and imprecision. The findings of this review are generalizable for LMICs in Asia since all the included studies were conducted in LMICs of Asia. We did not include stunting but rather BMI as a pre-specified primary outcome in this review since our target population was adolescents and not children. Moreover, only one of the included studies reported stunting as an outcome [31].

In light of the WHO building blocks framework, the service delivery platform in all the included studies was a school. The nutrition interventions were delivered through school teachers and student class monitors along with the study investigator. None of the included studies specified details pertaining to the health information system. In all of the included studies, the nutrition supplement was provided by the researcher, while financing was provided by various not-for-profit organizations. In all of the included studies, study investigators led the intervention.

The findings from this review should be interpreted with caution since the findings are based on few studies with high heterogeneity. We could not explore the possible causes of heterogeneity through subgroup or sensitivity analysis due to very few studies included in the review. We did not find any large-scale programs evaluating nutrition interventions among adolescents in LMICs. Two previous systematic reviews [19,20] assessed the effects of micronutrient supplementation. Both the reviews concluded that iron supplementation with or without folic acid reduces anemia, while our review findings are uncertain regarding any impact on anemia with iron supplementation with or without folic acid. The difference between these reviews and our review is that these reviews included youths along with the adolescents, while our review was restricted to the adolescent age group only. Many of the studies included in these reviews were excluded from our review due to the age cut-offs. Therefore, the number of eligible studies in these reviews was greater than our review, and our findings for anemia are based on a single study. Furthermore, these reviews included studies from upper-middle-income and high-income countries along with LMICs, while our review only included studies conducted in LMICs. One review [42] assessed the impact of micronutrient fortification. This review concluded that MMN fortification significantly improved anemia and hemoglobin levels; however, this review also included overlapping age groups of children and adolescents, as well as studies from upper-middle-income and high-income countries.

## 5. Conclusions

The evidence on preventive nutrition interventions among adolescents from LMICs is too scarce for any conclusive implications for practice. The existing evidence is limited to micronutrient supplementation/ fortification only, while there is no evidence on nutrition education and counseling and macronutrient supplementation among adolescents. Future studies assessing preventive nutrition interventions among adolescents should focus on assessing the effectiveness of nutrition education and macronutrient supplementation. There is a lack of focus on LMICs for this critical age group. Future studies should be well-designed with appropriate follow-up periods. Large-scale nutrition intervention program evaluations are needed in LMIC settings.

## Figures and Tables

**Table 1:** Characteristics of the included studies.

Study	Study Design	Setting	Participants	Intervention/Control	Outcomes
Agarwal, 2003 [27]	Cluster randomized trial	Four government senior secondary schools, Delhi, India	2088 adolescent girls	100 mg elemental iron and 500 μg folic acid in the form of oral tablets was provided for 100 days Group 1: Daily treatment (*N* = 702) Group 2: Weekly treatment: (*N* = 695) Control: Control group did not receive any tablets during the intervention period (*N* = 691)	Hemoglobin, plasma ferritin, anemia
Chiplonkar, 2012 [28]	Individually randomized trial	A secondary girl’s school in Pune City, Maharashtra, India	180 adolescent girls	Intervention group 1 (*N* = 60) Supplement was provided in the form of six different snacks to each girl with one snack (average amount 100 g/serving) per day for 6 school days in a week. The average zinc content of the food supplements was 2.2 ± 0.4 mg/serving Intervention group 2 (*N* = 59) The ayurvedic zinc tablet containing 20 mg of jasad bhasma, equivalent to 16.6 mg of elemental zinc, was given to each girl every day for 6 school days/week under the guidance of an ayurvedic doctor Control (*N* = 53) No supplement was given to the control group	Dietary intake, hemoglobin, plasma zinc, plasma beta-carotene, plasma retinol, plasma vitamin C
Februhartanty, 2002 [29]	Individually randomized trial	Junior high schools in Kupang, East Nusa Tenggara, in the eastern part of Indonesia	150 female adolescents	The iron tablet used in this study contained 60 mg elemental iron and 0.25 mg folic acid in the form of 200 mg ferrous sulfate Group 1: Weekly iron tablets (*N* = 50). Group 2: Iron tablet for four consecutive days during their menstruation cycle (*N* = 50) Control: Placebo tablet (*N* = 50)	Hemoglobin, ferritin level
Goyle, 2012 [30,37]	Individually randomized trial	Government school near university of Rajasthan, Jaipur, India	107 adolescent girls	Intervention group (*N* = 53): 100 g of biscuits fortified with one RDA levels of vitamin A, iron, folic acid, vitamin C, and iodine were provided for all working days during 4 months Control (placebo) (*N* = 54): 100 g of biscuits furnishing 497 kcal and 11.36 g of protein per day were provided to the control group for 4 months	Body mass index (BMI), BMI *Z*-score, weight-for-height, height-for-age
Hettiarrachchi, 2007 [31]	Individually randomized trial	School in the Galle district, Sri Lanka	821 school children	Children were supplemented with two capsules per day containing the following: Group 1: Iron (50 mg/day) in the form of ferrous fumarate (*N* = 202) Group 2: Zinc (14 mg/day) in the form of zinc sulfate (*N* = 213) Group 3: Combined (iron + zinc) (*N* = 216) Group 4: Placebo made of anhydrous lactose (*N* = 190)	Height, weight, body mass index (BMI), height-for-age, weight-for-age, stunted, underweight, hemoglobin, serum zinc, serum ferritin
Hyder, 2007 [35]	Individually randomized trial	Conducted in 54 non-formal primary education schools operated by the Bangladesh Rural Advancement Committee (BRAC) in Sherpur district, Dhaka	1125 adolescent girls	Group 1 (*N* = 559): Powdered beverage fortified with multiple micronutrients and packaged in sachets Control (*N* = 566): Placebo beverage	Weight, height, mid-upper arm circumference (MUAC), body mass index (BMI), hemoglobin, serum ferritin, serum retinol, serum zinc
Khadilkar, 2010 [32]	Individually randomized trial	State run school in Pune, India	50 adolescent girls	Group 1 (*N* = 25): Subjects in the treatment group were administered 6 vitamin D2 (ergocalciferol; Celltech, UK) tablets each containing 1.25 mg (50,000 IU) orally at 1, 4, 7, and 10 months Group 2 (*N* = 25): Placebo group; the local pharmacist prepared tablets containing only sucrose	Total body bone mineral content, lumbar spine bone mineral content and lumbar spine bone mineral apparent density, total body lean, fat mass, and serum concentrations of biochemical parameters
Sen, 2009 [33,38,39]	Cluster randomized trial	Municipal primary schools in Vadodara, India	358 girls	Group 1 (*N* = 94): The participants were given IFA tablets (100 mg elemental iron + 0.5 mg folic acid) once weekly Group 2 (*N* = 118): The participants were given IFA tablets (100 mg elemental iron + 0.5 mg folic acid) twice weekly Group 3 (*N* = 81): The participants were given IFA tablets (100 mg elemental iron + 0.5 mg folic acid) daily Group 4 (*N* = 65): Control group did not receive any intervention	Hemoglobin, body mass index (BMI), cognitive test score)
Soekarjo, 2004 [36]	Cluster randomized trial	Schools in Indonesia from both urban and rural locations	5166 adolescents aged 12–15 years	Group 1 (*N* = 1033): weekly 10,000 IU vitamin A Group 2 (*N* = 1045): weekly 60 mg elemental iron (as ferrous sulfate) plus 250 mg folate Group 3 (*N* = 1130): weekly 10,000 IU vitamin A and 60 mg elemental iron plus 250 mg folate Group 4 (*N* = 1958): Did not receive any supplement	Hemoglobin concentration, serum retinol concentrations
Zhu, 2005 [34,40,41]	Individually randomized trial	Schools in urban Beijing, China	757 adolescent girls	Group 1 (*N* = 238): Girls consumed a carton of 330 mL milk fortified with Ca on school days over the study period Group 2 (*N* = 260): Girls received the same quantity of milk additionally fortified with 5 or 8 mg cholecalciferol Group 3 (*N* = 259): Control girls did not receive any intervention	Nutrient intake, bone mineral content, bone mineral density, serum PTH, serum calcium, height, weight and vitamin D levels

**Table 2:** World Health Organization (WHO) health system building blocks framework.

Studies	Service Delivery	Health Workforce	Information Systems	Access to Supplies	Financing	Leadership
Agarwal, 2003	Delivery of iron supplements in school	Probably through school teachers	Not specified	Iron/folate supplements were provided by researchers	UNICEF, New Delhi	Researchers
Chiplonkar, 2012	Delivery of food supplements and zinc tablets in school	Probably through school teachers	Not specified	Food supplements and zinc tablets provided by researchers	Zensar Foundation, Pune, India	Researchers
Februhartanty, 2002	Delivery of iron supplements in schools	Delivered through school teachers	Not specified	Iron supplements were provided by researchers	SEAMEO-TROPMED Regional Center for Community Nutrition in Jakarta	Researchers
Goyle, 2012	Supplement biscuits in schools	Probably through school teachers	Not specified	Biscuits were supplied through researcher	University Grants Commission, New Delhi, India	Researchers
Hettiarachchi, 2007	Iron and zinc supplements provided in schools	Delivered through teachers and investigators	Not specified	Supplements were provided by the researchers	The study was funded by the International Atomic Energy Agency	Researchers
Hyder, 2007	Iron fortified beverage provided in school	Delivered through school teachers with the assistance of the Bangladesh Rural Advancement Committee (BRAC) community health workers	Not specified	Supplements were provided by the researchers	Supported by the Micronutrient Initiative, Ottawa, Canada	Bangladesh Rural Advancement Committee (BRAC)
Khadilkar, 2010	Vitamin D supplements were provided in school	The tablets were supplied to participants monthly by trial staff	Not specified	Supplements were provided by the researchers	Not specified	Researchers
Sen, 2009	Iron/folic acid supplements were provided in schools	Investigators, monitors, class teachers	Not specified	Supplements were provided by the researchers	None	Researchers
Soekarjo, 2004	Vitamin A, iron, and folate supplements were provided in the schools	Field workers supervised the supplement intake	Not specified	Supplements were produced locally and provided by the researcher	This study was funded by USAID through the OMNI project	Researchers
Zhu, 2005	Milk supplementation given in schools	Probably through school teachers	Not specified	Milk supplementation given in schools	Australian Dairy Research and Development Corporation, Murray Goulburn Co-operative Co. Limited, and the Nestle’ Foundation provided financial support for the laboratory analyses	Researchers

**Table 3:** Summary of findings.

**Patient or Population**: Adolescents **Settings**: Schools **Intervention**: Micronutrient supplementation/fortification **Comparison**: Placebo/no supplementation/no fortification					
**Outcomes**	Illustrative Comparative Risks * (95% CI)		**Relative Effect (95% CI)**	**No of Participants (Studies)**	**Quality of the Evidence (GRADE)**
	**Assumed Risk**	**Corresponding Risk**			
	**Placebo/No Supplementation**	**Micronutrient Supplementation/Fortification**			
**Daily Iron Supplementation with or without Folic Acid: Anemia**	Study population		RR 1.04 (0.88 to 1.24)	1160 (one study)	⊕⊕⊝⊝ low ^1,2^
	206 of 579	216 of 581			
**Weekly Iron Supplementation with or without Folic Acid: Anemia**	Study population		RR 1.07 (0.91 to 1.26)	1274 (one study)	⊕⊕⊝⊝ low ^1,2^
	206 of 579	265 of 695			
**Calcium/Vitamin D Supplementation/Fortification: BMI**	Study population		MD −0.01 (−1.2 to 1.17)	730 (2 studies)	⊕⊝⊝⊝ very low ^1,2,3^
	The mean BMI ranged between 18.15 and 18.5	The mean BMI ranged between 17.05 and 19.1			
**Iron Supplementation with or without Folic Acid: BMI**	Study population		MD 0.29 (−0.25 to 0.83)	652 (2 studies)	⊕⊝⊝⊝ very low ^1,2,3^
	The mean BMI ranged between 15.78 and 16.23	The mean BMI ranged between 15.67 and 17.25			
**Zinc Supplementation: BMI**	Study population		MD 0.35 (−0.15 to 0.85)	382 (one study)	⊕⊝⊝⊝ very low ^1,2,3^
	The mean BMI was 16.23	The mean BMI was 16.58			
**MMN Fortification: BMI**	Study population		MD 0.23 (−0.11 to 0.57)	943 (2 studies)	⊕⊝⊝⊝ very low ^1,2,3^
	The mean BMI ranged between 15.27 and 16.5	The mean BMI ranged between 15.42 and 17.1			

* The basis for the assumed risk (e.g., the median control group risk across studies) is provided in the footnotes. The corresponding risk (and its 95% confidence interval) is based on the assumed risk in the comparison group and the relative effect of the intervention (and its 95% CI). CI: confidence interval; RR: risk ratio; MD: mean difference. GRADE: Grading of Recommendations, Assessment, Development, and Evaluation. High quality: further research is very unlikely to change our confidence in the estimate of effect. Moderate quality: further research is likely to have an important impact on our confidence in the estimate of effect and may change the estimate. Low quality: further research is very likely to have an important impact on our confidence in the estimate of effect and is likely to change the estimate. Very low quality: we are very uncertain about the estimate. ^1^ Downgraded by one level due to study limitations. ^2^ Downgraded by one level due to imprecision. ^3^ Downgraded by one level due to high heterogeneity.

## Supplementary Materials

The following are available online at https://www.mdpi.com/2072-6643/12/1/49/s1, Table S1: PRISMA Checklist.

## Appendix A. Search Strategy

**PubMed Search Strategy: (Titles/Abstracts and Text Words)**	((“Adolescent” [Mesh]) OR (“Child” [Mesh]) OR (Adolescent* OR Adolescence)OR (Teen* OR (Youth*) OR (Puberty) OR (juvenil*)) AND ((“Micronutrients” [Mesh]) OR (“Dietary Supplements” [Mesh]) OR (“Food, Fortified” [Mesh]) OR (“Vitamins” [Mesh]) OR (“Minerals” [Mesh] OR “Trace Elements” [Mesh]) OR (“Ferric compounds” [Mesh] OR “Ferrous Compounds” [Mesh]) OR (Iron* OR Ferric OR Ferrous) OR (“Diet Supplement*” OR “Dietary Supplement*” OR Biofortification) OR (“Folic Acid” [Mesh]) OR (Folic* OR Folate* OR Folvite* OR Folacin*) OR (“Zinc” [Mesh] OR “Zinc Sulfate” [Mesh]) OR (“Calcium” [Mesh]) OR (Calcium) OR (“Vitamin D” [Mesh]) OR (vitamin d) OR (“Vitamin A” [Mesh]) OR (“Vitamin A”) OR (“Ascorbic Acid” [Mesh]) OR (“Vitamin C”) OR (Ascorb* OR “ascorbic acid”) OR (Vitamin* OR multivitamin* OR multi-vitamin* OR MMN OR micro-nutrient* OR mineral* OR multimineral* OR multi-mineral OR multinutrient* OR “multiple micronutrient*” OR “food environment” OR advertisement* OR “mass media” OR “supplementary feeding” OR “energy supplement*” OR “protein supplement*” OR “lipid based nutrition” OR LNS)) AND ((“Adolescent Development” [Mesh]) OR (“Adolescent Growth”) OR (“Serum Haemoglobin” OR “Serum micronutrient*” OR “Anthropometric measurement*”))
**EBSCO CINAHL Plus:**	((“Adolescent” [Mesh]) OR (Adolescent* OR Adolescence) OR (Teen* OR Teenager*) OR (Youth*) OR (Puberty) OR (juvenile)) AND ((“Micronutrients” [Mesh]) OR (“Dietary Supplements” [Mesh]) OR (“Food, Fortified” [Mesh]) OR (“Vitamins” [Mesh]) OR (“Minerals” [Mesh] OR “Trace Elements” [Mesh]) OR (“Ferric compounds” [Mesh] OR “Ferrous Compounds” [Mesh]) OR (Iron* OR Ferric OR Ferrous) OR (“Diet Supplement*” OR “Dietary Supplement*” OR Biofortification) OR (“Folic Acid” [Mesh]) OR (Folic* OR Folate* OR Folvite* OR Folacin*) OR (“Zinc” [Mesh] OR “Zinc Sulfate” [Mesh]) OR (“Calcium” [Mesh]) OR (Calcium) OR (“Vitamin D” [Mesh]) OR (vitamin d) OR (“Vitamin A” [Mesh]) OR (“Vitamin A”) OR (“Ascorbic Acid” [Mesh]) OR (“Vitamin C”) OR (Ascorb* OR “ascorbic acid”) OR (Vitamin* OR multivitamin* OR multi-vitamin* OR MMN OR micro-nutrient* OR mineral* OR multimineral* OR multi-mineral OR multinutrient* OR “multiple micronutrient*” OR “food environment” OR advertisement* OR “mass media” OR “supplementary feeding” OR “energy supplement*” OR “protein supplement*” OR “lipid based nutrition” OR LNS)) AND ((“Adolescent Development” [Mesh]) OR (“Adolescent Growth”) OR (“Serum Haemoglobin” OR “Serum micronutrient*” OR “Anthropometric measurement*”))
**Cochrane Library:**	((“Adolescent” [Mesh]) OR (“Child” [Mesh]) OR (Adolescent* OR Adolescence) OR (Teen* OR (Youth*) OR (Puberty) OR (juvenil*)) AND ((“Micronutrients” [Mesh]) OR (“Dietary Supplements” [Mesh]) OR (“Food, Fortified”[Mesh]) OR (“Vitamins” [Mesh]) OR (“Minerals” [Mesh] OR “Trace Elements” [Mesh]) OR (“Ferric compounds” [Mesh] OR “Ferrous Compounds” [Mesh]) OR (Iron* OR Ferric OR Ferrous) OR (“Diet Supplement*” OR “Dietary Supplement*” OR Biofortification) OR (“Folic Acid” [Mesh]) OR (Folic* OR Folate* OR Folvite* OR Folacin*) OR (“Zinc” [Mesh] OR “Zinc Sulfate” [Mesh]) OR (“Calcium” [Mesh]) OR (Calcium) OR (“Vitamin D” [Mesh]) OR (vitamin d) OR (“Vitamin A” [Mesh]) OR (“Vitamin A”) OR (“Ascorbic Acid” [Mesh]) OR (“Vitamin C”) OR (Ascorb* OR “ascorbic acid”) OR (Vitamin* OR multivitamin* OR multi-vitamin* OR MMN OR micro-nutrient* OR mineral* OR multimineral* OR multi-mineral OR multinutrient* OR “multiple micronutrient*” OR “food environment” OR advertisement* OR “mass media” OR “supplementary feeding” OR “energy supplement*” OR “protein supplement*” OR “lipid based nutrition” OR LNS)) AND ((“Adolescent Development” [Mesh]) OR (“Adolescent Growth”) OR (“Serum Haemoglobin” OR “Serum micronutrient*” OR “Anthropometric measurement*”))

## Appendix B. Reasons for Exclusion

**Study**	**Reason for Exclusion**
Abrams, 2005 [43]	Intervention given was prebiotic (inulin-type fructans)
Ahmed, 2005 [44]	Participants were anemic at baseline and the intervention was therapeutic
Ahmed, 2010 [45]	Participants were anemic at baseline and the intervention was therapeutic
Angeles-Agdeppa, 1997 [46]	Participants were asymptomatic anemic individuals and the intervention was therapeutic
Beasley, 2000 [47]	Participants were infected with schistosomiasis; infection was believed to affect the outcome, and IFA was taken as therapeutic intervention
Castillo-Durán, 2001 [47]	The study was from a non-LMIC country
Chan, 2006 [48]	The study was carried out in a non-LMIC country
Damsgaard, 2012 [49]	Only slightly overweight individuals were enrolled
de Oliveira, 2009 [50]	The study was from a non-LMIC country
Deshmukh, 2008 [51]	This study did not have an appropriate control group
Diogenes, 2013 [52]	The study was from a non-LMIC country
Dongre, 2011 [53]	This study did not have an appropriate control group
Eftekhari, 2006 [54]	Participants were iron-deficient at baseline and the intervention was therapeutic
Friis, 1997 [55]	93% of the participants were infected by schistosomiasis; infection was believed to affect the outcome
Ganmaa, 2017 [56]	Participants were asymptomatic vitamin D-deficient individuals according to the inclusion criteria; intervention was used as therapeutic intervention
Ilich-Ernst, 1998 [57]	The study was carried out in a non-LMIC country
Kianfer, 2000 [58]	The intervention was therapeutic
Kotecha, 2009 [59]	The study did not have an appropriate control group
Lambert, 2009 [60]	The study was carried out in a non-LMIC country
Ma, 2014 [61]	The study did not have an appropriate control group
Manger, 2008 [62]	The study population included children and adolescents, and the study author suggested that the data for the adolescent subgroup were too small
Mann, 2002 [63]	Participants were asymptomatic anemic individuals; grouping was done based on energy intakes
McKenna, 1997 [64]	The study was carried out in a non-LMIC country
Mwaniki, 2002 [65]	The intervention was therapeutic
Pilz, 2017 [66]	The methods described inclusion criteria of age 18–45 years but results showed that the age of participants was between 22 and 29 years; participants were not adolescents
Prentice, 2005 [67]	The study was carried out in a non-LMIC country
Dibba, 2000 [68]	The study population included children and adolescents; the corresponding authors were contacted for the adolescent subgroup data; however, we did not receive any response
Rerksuppaphol, 2016 [69]	The study population included children and adolescents; the corresponding authors were contacted for the adolescent subgroup data; however, we did not receive any response
Rousham, 2013 [70]	Intervention was used as therapeutic intervention
Sarma, 2006 [71]	The study population included children and adolescents; the corresponding authors were contacted for the adolescent subgroup data; however, we did not receive any response
Schou, 2003 [72]	The study was carried out in a non-LMIC country
Shah, 2002 [73]	Intervention was used as therapeutic intervention
Silk, 2015 [74]	The study was carried out in a non-LMIC country
Sunawang, 2009 [75]	The participants were not adolescents
Tee, 1999 [76]	Participants were asymptomatic anemic individuals; intervention was used as a therapeutic agent in this study
Viljakainen, 2006 [77]	The study was carried out in a non-LMIC country
White, 2015 [78]	The study was carried out in a non-LMIC country
Yusoff, 2012 [79]	The study was from a non-LMIC country
